# Biomolecule-Mediated Synthesis of Selenium Nanoparticles using Dried *Vitis vinifera* (Raisin) Extract

**DOI:** 10.3390/molecules19032761

**Published:** 2014-02-27

**Authors:** Garima Sharma, Ashish Ranjan Sharma, Riju Bhavesh, Jongbong Park, Bilguun Ganbold, Ju-Suk Nam, Sang-Soo Lee

**Affiliations:** 1 Amity Institute of Nanotechnology, Amity University Uttar Pradesh, Noida, Uttar Pradesh 201303, India; 2 Institute for Skeletal Aging & Orthopaedic Surgery, Chuncheon Sacred Heart Hospital, College of Medicine, Hallym University, Chuncheon 200704, Korea

**Keywords:** *Vitis vinifera*, selenium, lignin, FTIR, EDX, nanoparticles, XRD, biosynthesis

## Abstract

Biomolecule-mediated nanoparticle synthesis has recently the gained attention of researchers due to its ecofriendly and non-toxic nature. Metabolites from plant extracts represent a better alternative to chemical methods to fulfill the growing demand for non-hazardous nanoparticle synthesis routes. Selenium and its nanoparticles have an extensive range of applications. Thus, biofabrication of selenium nanoparticles can be potentially useful in various fields. This study reports a green approach to biosynthesize selenium nanoparticles (Se-np) using dried *Vitis vinifera* (raisin) extracts. The biosynthesized selenium nanoparticles were characterized using transmission electron microscope (TEM), dynamic light scattering (DLS), X-ray diffraction (XRD), energy dispersive X-ray (EDX) spectroscopy and Fourier transform infrared spectroscopy (FTIR). Transmission electron microscopic images revealed the spherical shape of biosynthesized selenium nanoparticles and a size range of 3–18 nm. Dynamic light scattering also confirmed the average particle size of 8.12 ± 2.5 nm with 0.212 PDI. The crystalline nature of selenium nanoparticles was confirmed by the X-ray diffraction study. Moreover, as inferred from the FTIR spectrum, the presence of highly stable lignin biopolymer on the surface of selenium nanoballs suggests a possible role as capping agent.

## 1. Introduction

In recent years, interest in nanoparticles and nanomaterials, with sizes ranging from 0.1 nm to 1,000 nm, has emerged due to their novel and enhanced applicability in various areas such as electronics, chemistry, energy, and the development of medicines [1]. Today, various chemical, physical and biological methods for nanoparticle synthesis are used, among which biosynthetic approaches are considered as ecofriendly and economic for many reasons [2]. Bio-macromolecules can serve as a potential biocatalyst in producing a wide range of nanomaterials. Besides working as bioreducing agents, they can additionally act as natural stabilizers for nanoparticles. Thus, biogenic material-mediated nanoparticle synthesis reduces the use of toxic chemicals and leads to an ecofriendly green synthesis of nanoparticles [3]. Biosynthesis of nanoparticles can be performed using various biomaterials like bacteria, fungus, algae and plants. Among these biomaterials plant extract-mediated extracellular nanoparticle synthesis is of great interest as it is rapid, easy and does not require special conditions. Furthermore, due to absence of toxic chemicals, biosynthesized nanoparticles are suitable for biological applications [4]. Thus, in this study dried *vitis vinefera* (raisin) extract was employed as a substrate for nanoparticle synthesis. Raisin contains sugars (~60%), flavonoids, phenolic compounds, minerals, iron, vitamins, potassium, calcium, *etc.* [5], which might facilitate the synthesis of selenium nanoparticles.

Selenium (Se) is a semiconductor used in many applications like production of photovoltaic cells, rectifiers, photographic exposure meters, xenography, *etc.* [6]. It is also one of the essential trace minerals which incorporates into proteins to prevent cellular damage, regulates the function of the thyroid gland and helps in the proper functioning of the immune system [7]. Additionally, selenium also serves as a strong antimicrobial and anti-carcinogenic agent against a variety of cancers [8,9]. Despite these various advantages, high doses of selenium can cause adverse effects. Therefore, studies are now focusing on how to overcome the drawbacks of high doses of selenium by using its nanoparticles, while maintaining the biological effects such as the anticancer activity [10]. Recent reports have revealed that selenium nanoparticles possess increased biological activity with reduced risk of selenium toxicity [11]. Although various physicochemical methods are used for selenium nanoparticle synthesis, there has been increasing interest in the synthesis of selenium nanoparticles using biological approaches leading to the development of non-toxic and environmentally friendly biomimetic systems [12]. Biosynthesis of selenium nanoparticles using microorganisms and plant parts have previously been reported in some studies [13,14]. The use of plant extracts for the synthesis of nanomaterials is cost competitive over the use of fungal and bacterial broth as it avoids the cost of microorganism isolation and culture media.

In the present study, an ecofriendly biosynthesis of selenium nanoparticles using *Vitis vinifera* (raisin) extract is reported. The synthesis method is free of any toxic reducing agents and organic solvents. The biosynthesized selenium nanoparticles are further characterized by different techniques.

## 2. Results and Discussion

### 2.1. Atomic Absorption Spectroscopy Analysis

**Figure 1:**
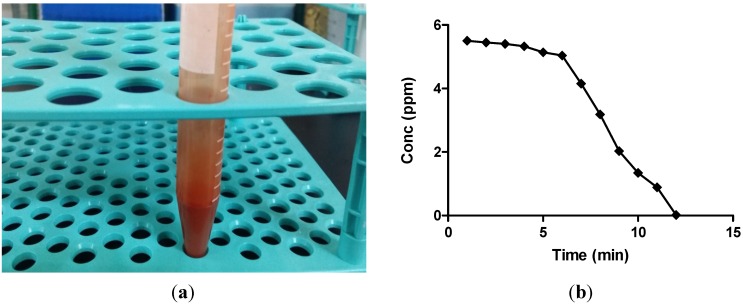
(**a**) Photograph of biosynthesized selenium nanoparticles; (**b**) AAS graph of selenium ion concentration.

Biogenic synthesis of selenium nanoparticles was confirmed by the conversion of colourless selenious acid into the brick red colour of selenium nanoparticles (Figure 1a). Further, to investigate the conversion of selenium ions into selenium nanoballs atomic absorption spectroscopy (AAS) was used. For this, a standard solution of selenious acid (5.5 ppm) was prepared and was normalized at 0 min for further readings. Samples were withdrawn at various time intervals throughout the reaction and were centrifuged. Supernatant was analyzed by AAS for the remaining selenium ion concentration. Selenium nanoballs tend to precipitate at 14,500–15,000 rpm, whereas selenium ions, being much smaller in size, stay in the solution. Figure 1b shows the decrease in selenium ion concentration from an initial value of 5.5 ppm to a final value of 0.02 ppm after 12 min of reaction time, hence showing the conversion of selenium ions into selenium nanoparticles. Moreover, it also shows that the concentration of selenium ions was almost constant up to the first 6 min, which indicates that the conversion of selenium ions into selenium nanoparticles starts around 6 min and further continues up to 12 min.

### 2.2. Morphology and Size Analysis

**Figure 2:**
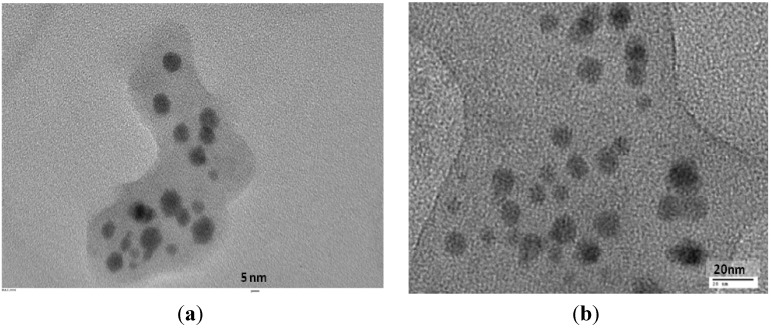
TEM images of biofabricated selenium nanoballs using *Vitis vinifera* extract (**a**) at the scale of 5 nm; (**b**) at scale of 20 nm.

**Figure 3:**
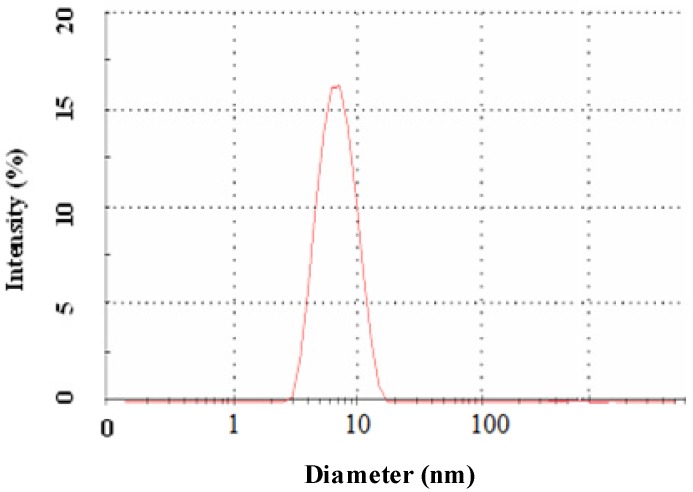
Size distribution pattern of biosynthesized selenium nanoparticles.

Further, the size and morphology of the biosynthesized selenium nanoparticles were characterized. TEM images of the prepared selenium nanoparticles showed a uniform distribution and confirmed their spherical morphology. The depicted ball-like structure of the selenium nanoparticles in the TEM images ranged in size from 3–18 nm in diameter. Figure 2a,b shows selenium nanoparticles at 5 nm and 20 nm scale, respectively. In the figures a thin film encapsulating the nanoballs is seen, confirming the presence of a polymeric layer covering the nanoballs. Figure 3 shows the DLS measurement of the hydrodynamic effective diameters of the biosynthesized selenium nanoballs [15]. The zeta average diameter was measured to be 8.12 ± 2.5 nm with 0.212 PDI. The disagreement between the sizes observed by TEM and DLS was due to the fact that DLS measures the hydrodynamic volume while TEM analyzes the metallic core [16].

### 2.3. Energy Dispersive X-ray Analysis

**Figure 4:**
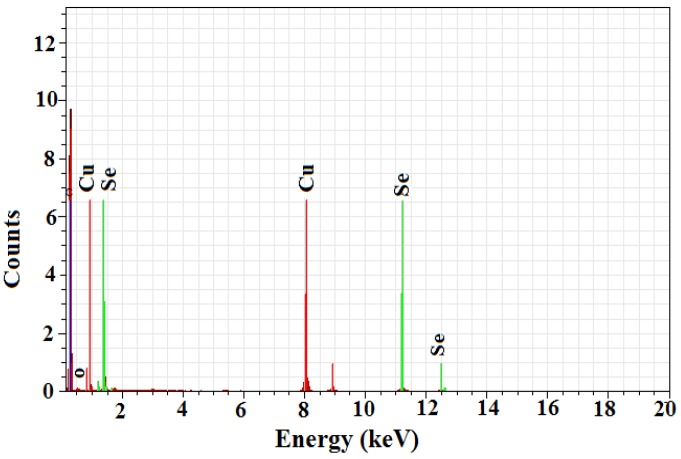
EDX spectroscopy of biofabricated selenium nanoballs.

The energy dispersive X-ray spectroscopy analysis confirmed the presence of elemental selenium nanoparticles in the given sample, as depicted by Figure 4. The selenium nanoballs showed characteristic absorption peaks of selenium at 1.37 keV (SeLα peak), 11.22keV (SeKα peak) and 12.49 keV (SeKβ peak) [17]. The maximum peak located on the left part of the spectrum at around 0.2 kV clearly indicates the presence of carbon, while the hardly visible maximum located at 0.5 keV is associated with the characteristic oxygen line. Copper peaks were also visible in the EDX spectra which were due to the Cu support grid. The lack of other elemental peaks and high amount of selenium in the spectra confirms the purity of the selenium metal in the transformed product. The presence of carbon and oxygen spots in the samples confirms the presence of stabilizers composed of alkyl chains.

### 2.4. X-ray Diffraction Analysis

**Figure 5:**
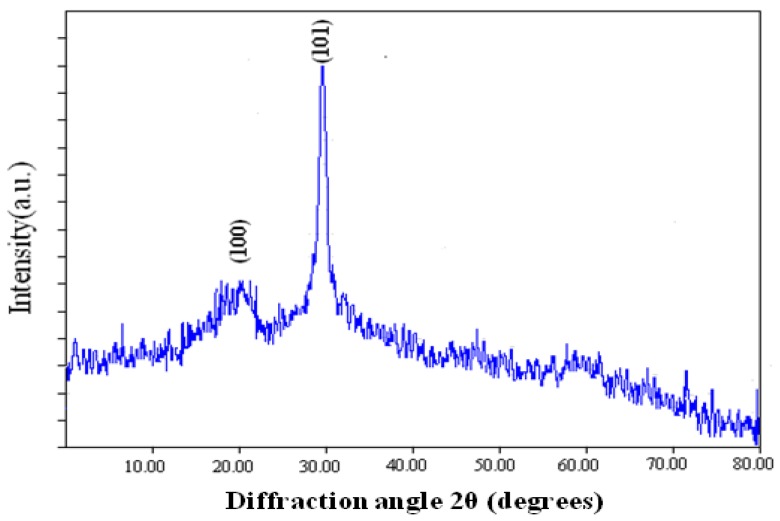
XRD pattern of biofabricated selenium nanoball.

The crystalline nature of the selenium nanoballs was analyzed using X-ray diffraction. Figure 5 shows broad diffraction peaks at lower angles, hence confirming the amorphous/nano-crystalline nature of the sample [12]. The diffraction peaks were in accordance with the trigonal phase of selenium having lattice constants a = 4.362 Å and c = 4.958 Å, which corresponds to the reported value (JCPDS File No. 06-362). Full width at half maximum (FWHM) data was used to determine the particle size through Scherrer’s formula [18], and it was found to be 12 nm.

### 2.5. Fourier Transform Infrared Spectroscopy

**Figure 6:**
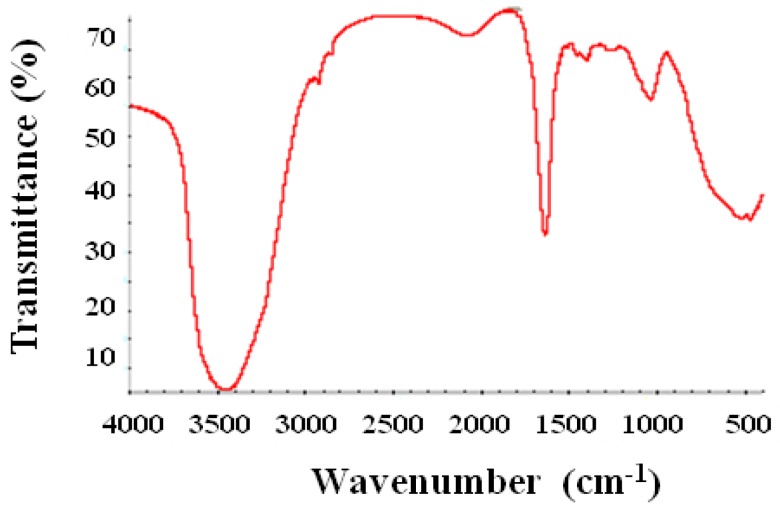
FTIR pattern of biofabricated selenium nanoballs.

**Figure 7:**
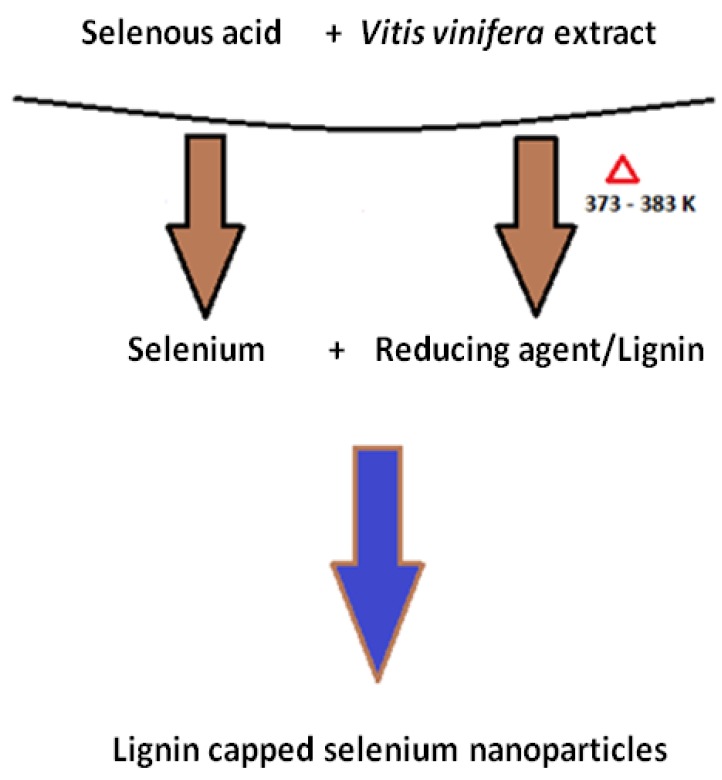
Hypothetical mechanism of selenium nanoparticle synthesis using *Vitis vinifera* extract.

The synthesized selenium nanoballs were characterized using FTIR in order to investigate the biological compounds responsible for the synthesis and stability of the particles. The result (Figure 6) shows sharp absorption peaks at 3420 cm^−1^ and 1620 cm^−1^. Peak at 3420 cm^−1^ can be assigned to OH and the one at 1620 cm^−1^ corresponds to the C-H vibration of the aromatic ring. Apart from these two prominent peaks, there are peaks at 1375 cm^−1^, 1030 cm^−1^, 1462 cm^−1^ and 1250 cm^−1^ representing phenolic OH, aromatic in-plane C-H bending, asymmetric C-H bending (in CH_3_ and -CH_2_-) and secondary OH, respectively. Peaks at 2840 and 2930 cm^−1^ represent ether-methoxy-OCH_3_ groups. These peaks show the presence of the biopolymer lignin associated with the selenium nanoparticles [19,20,21,22]. Lignin is a biopolymer found in vegetables, fruits and the secondary cell walls of plants which can be easily extracted from woods [23]. The result indicates the presence of lignin (a phenolic compound) as a bio-polymeric agent which may be responsible for both the reduction and stabilization of the selenium nanoballs. Previous reports have also suggested the role of lignin as a reducing and stabilizing agent for the synthesis of some metal nanoparticles [24]. The high glucose and fructose content in dried *Vitis vinifera* might also act as reducing sugars during the synthesis of selenium nanoparticles [25]. However, the identity of the molecules responsible for the synthesis and stabilization of the selenium nanoparticles need further validation. The hypothetical mechanism of Se nanoball synthesis is shown in Figure 7.

## 3. Experimental

### 3.1. Synthesis of Selenium Nanoparticles

Selenous acid (H_2_SeO_3_) was purchased from Sigma-Aldrich (St. Louis, MO, USA). Shade dried *V. vinifera* fruits (5 g, purchased from an ayurvedic shop in New Delhi, India) were soaked overnight and crushed finely. For extract preparation the finely crushed *V. vinifera* fruits were refluxed for 30 min in distilled water. The extract obtained was filtered twice with Whatman paper No.1 and stored at 4 °C till further use. Extract (10 mL) was added to solution of 4 × 10^−5^ M selenous acid (90 mL) and refluxed for 15 min. Selenium nanoparticles were centrifuged and precipitated at 15,000 rpm.

### 3.2. Characterization of Selenium Nanoparticles

#### 3.2.1. Atomic Absorption Spectroscopy

Atomic absorption spectroscopy (GBC 932 AA, Braeside, Australia) was performed using an air/acetylene flame to observe the conversion of selenium ions from selenous acid into zero-valent selenium nanoparticles [26]. Briefly, at the start acalibration curve was established with AAS measurement of various selenium ion solutions of known concentrations which was further used to quantitatively determine the selenium ion concentration in the reaction sample at predetermined time points during the reaction. For accuracy, the experiment was performed in triplicate.

#### 3.2.2. Transmission Electron Microscopy

The morphology and size of selenium nanoparticles were characterized using transmission electron microscopy (TEM, Philips CM 200, Hillsboro, OR, USA) operating at an accelerating voltage of 200 kV. The reaction solution was diluted with deionized water and sonicated (Vibronics VS 80, Mumbai, India) for 10 min. The sonicated sample was drop coated on carbon coated copper grids and vacuum dried for half an hour and the electron micrographs were taken.

#### 3.2.3. Dynamic Light Scattering Spectroscopy

For analysis of the particle size distribution in the sample, dynamic light scattering (DLS, Zetasizer Nano ZS ZEN 3600, Malvern, UK) was used at 25 °C using automatically adjusted attenuation laser filters. The study revealed the size and PDI of the nanoballs. The particle size was given as mean ± SD (n = 3).

#### 3.2.4. Energy Dispersive X-ray Study

Energy Dispersive X-ray (EDX) was done to confirm the conversion of selenium ions into elemental selenium (Se) using a Philips CM200 200 kV TEM. The sample was prepared in a similar manner as that for TEM. The selected micrograph from TEM was then subjected to elemental analysis by EDX (Bruker Inc., Madison, WI, USA). The atom percentage of metal in EDX analaysis helps to determine the purity and elemental composition of the sample.

#### 3.2.5. X-ray Diffraction Study

For X-ray diffraction (XRD) measurement, drop coated film of biosynthesized selenium nanoballs on the glass substrate was analyzed with an εMMA Diffractometer (Braeside, Australia) operating at a voltage of 40 kV and current of 20 mA with Cu K(α) radiation of 1.54187 nm wavelength. The scanning was done in the 2θ range of 20° to 80° at 0.02°/min with time constant of 2 s.

#### 3.2.6. Fourier Transform Infrared Spectrpscopy

The Fourier transform infrared (FTIR) pattern revealed the possible biomolecules responsible for synthesis and stabilization of the nanoballs. For analysis, the sample was centrifuged at 14,000 rpm and the supernatant was replaced with deionized water to resuspend the nanoballs. This process was repeated thrice to ensure purity by complete removal of any unbound compounds. The precipitate was then vacuum dried and ground with KBr to form pellets which were then analyzed using a Nicolet IR 200 (Thermo Electron Corp, Madison, WI, USA).

## 4. Conclusions

Herein, we have reported an ecofriendly method of fabricating very small and uniformly shaped selenium nanoballs. In this biological and ecofriendly approach dried *Vitis vinifera* extract played a significant role in synthesizing biopolymer (lignin)-capped selenium nanoballs. The selenium nanoballs produced were of about 3–18 nm in size, as determined by TEM and DLS techniques. The synthesized selenium nanoballs were uniform in shape and size and can be exploited further for pharmaceutical application. The exact mechanism of selenium nanoparticle synthesis using *Vitis vinifera* extract and its applications are yet to be explored further.
